# The Treatment of Influenza

**Published:** 1890-03

**Authors:** 


### THE TREATMENT OF INFLUENZA.

M. Henri Huchars, writing in the *Revue generale de Clinique et de Therapeutique*, of December 12, 1889, offers some suggestions concerning the treatment of influenza which may be of interest at this time. "In all epidemics of the disease," he says, "the most marked clinical feature is the adynamic character of the symptoms, and it is against this that treatment should be chiefly directed. For this reason preparations of cinchona, alcoholic beverages, and the like should be prescribed early; and in grave cases, when the prostration is profound, injections of ether or of caffeine may be needed. Quinine is usually indicated by the remittent character of the fever, and, for the control of this, should be given in one dose of from eight to fifteen grains in the forenoon. If the fever persists in spite of this, it may be well to give fifteen grains of antipyrine in the evening. But even when the fever is not high the use of quinine in tonic doses is advisable. In the rheumatoid or neuralgic forms, characterized by severe pains in the joints, muscles, and other parts of the body, we may resort to antipyrine in doses of fifteen grains two or three times a day. Instead of this drug we may use phenacetine or salol in eight-grain doses. When the disease assumes the broncho-pulmonary form the adynamia is commonly very pronounced, and the necessity for stimulants becomes even more pressing. The nourishment should consist chiefly of milk, which acts as a diuretic, and favors the elimination of the waste-products. Blisters are inadmissible in the pneumonia following influenza, which is pre-eminently a local manifestation of a general disease, for they do not benefit the local process, and only aggravate the general condition. Occasionally the dyspnœa, even when the pulmonary affection is apparently limited in extent, becomes extreme. In this case much benefit is often derived from the use of strychnine. Sometimes, especially in the renal form of the affection, the only thing that will relieve the dyspnœa is a venesection, though ordinarily this is the last thing to be thought of in a disease in which the adynamic tendency is so pronounced. In the gastro-intestinal form it is necessary to employ mild purgatives, such as castor-oil or calomel, and to obtain intestinal antisepsis by means of salicylate of bismuth, or of magnesia, napthol, etc. If an emetic is at any time indicated, tartar emetic should never be used, but ipecac given in preference."—*Medical Record.*

